# A 110-year-old wise man: Professor Libin T. Cheng, one of the founders of biochemistry and nutrition in China

**DOI:** 10.1007/s13238-017-0428-0

**Published:** 2017-05-24

**Authors:** He Zhang

**Affiliations:** 1 Department for the History of Science and Scientific Archaeology, University of Science and Technology of China, Hefei, 230026 China; 2 School of Marxism, Bengbu Medical College, Bengbu, 233030 China

Do you believe in so-called food-combination poisoning? Do you have the courage to eat crab and Chinese persimmon together? In fact, there is no need to worry about poisoning. As early as 80 years ago, it was proved that so-called food-combination poisoning is a superstition, through experiments by a man named Libin T. Cheng (1900–2010), who was one of the founders of biochemistry and nutrition in China and who also became a 110-year-old wise man.

**Figure 1:**
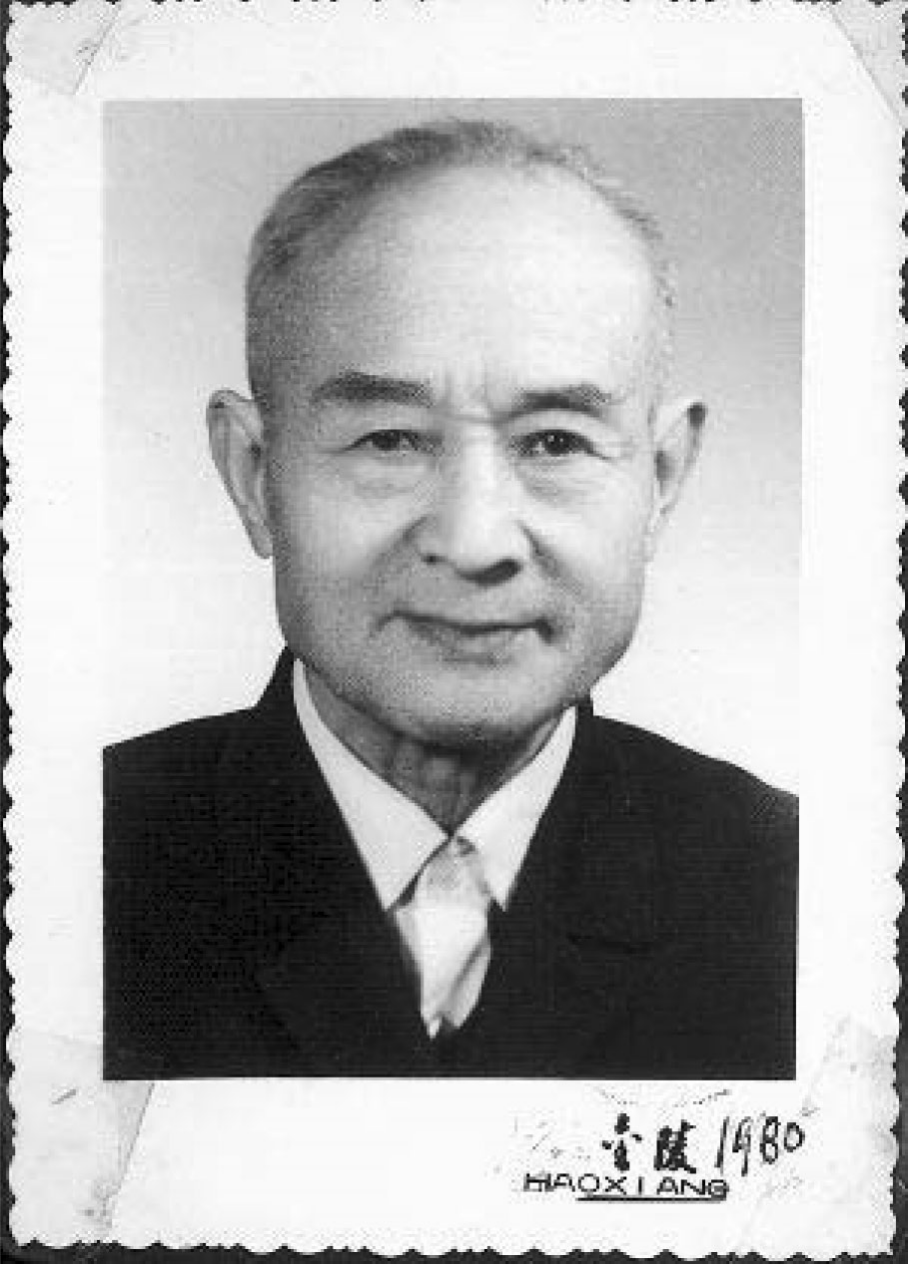
Professor Libin T. Cheng (1900–2010)

**Figure 2:**
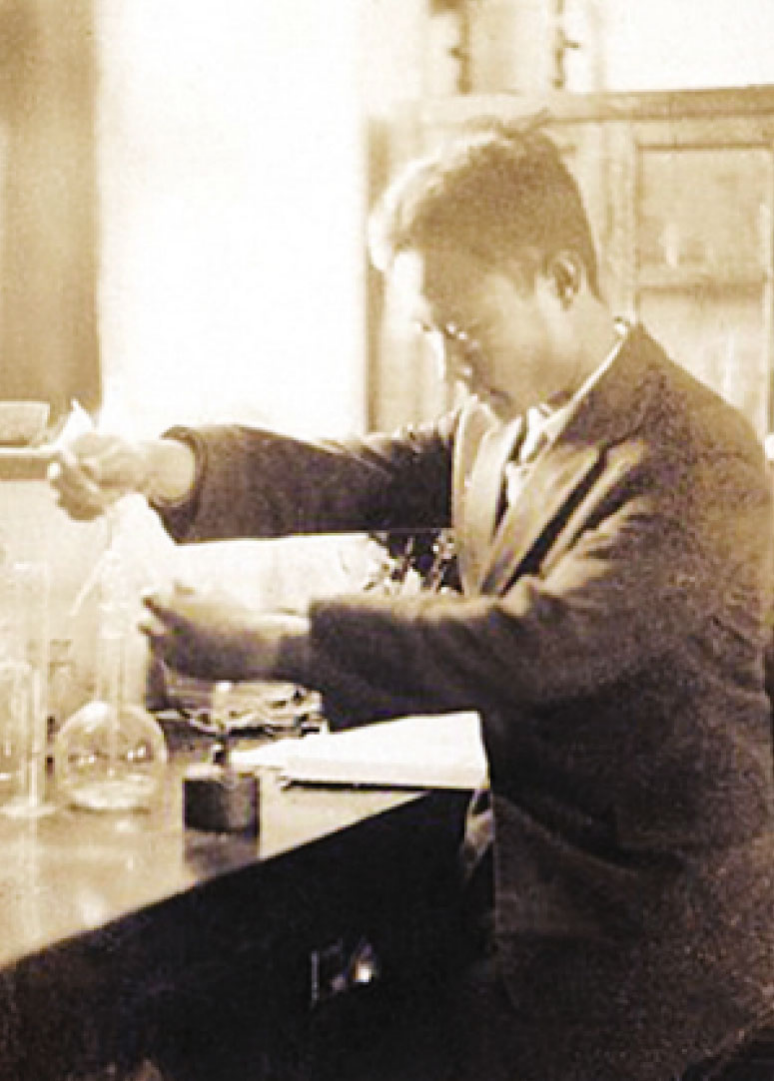
Libin T. Cheng at work in the Central Medical School in the 1930s

Libin T. Cheng (Fig. 1) was born in Nanxi County, Sichuan Province, in 1900. He received his bachelor’s degree from National Central University in 1928, master’s degree from Ohio State University in 1931, and doctor’s degree from Indiana University in 1934. In 1934, Cheng returned to China and took charge of establishing the Department of Physiological Chemistry in the Biological Laboratory of the Science Society of China, invited by Professor Bing Zhi (Li and Kang, 2010), a pioneer of modern biology in China. In 1936, Cheng was appointed as professor and director of the Department of Biochemistry in the Central Medical School of National Central University (Fig. 2). In 1945, he established a biochemistry research institute in the Central Medical School, which was the first formal organization to produce biochemistry postgraduates in the history of education in China. In 1957, Cheng established biochemistry as a major at Nanjing University. Two years later, he formally served as professor of the Department of Biology and director of the biochemistry teaching and research office. In 1934, Libin T. Cheng was selected as a member of the Sigma Xi Society; in 1956, he was awarded as the First Grade Professor; in 1996, he was selected as a member of the American Association for the Advancement of Science.

During his postgraduate education in the United States, Cheng studied at the University of Chicago and Yale University successively and had the opportunity of contact with the famous biochemists at that time, such as plant protein chemistry experts and nutritionists L. B. Mendel and H. B. Vickery, Vitamin B nutritionist G. R. Cowgill, and mineral nutritionist A. H. Smith. Because of a strong interest in protein chemistry and nutrition, he chose to conduct research on the extraction and physicochemical properties of soybean protein in the laboratory of R. T. Hartman at Indiana University, from which he received a doctor’s degree. One of his important reasons for choosing soybean protein as a research subject was that it was the main source of protein in Chinese people’s diets at that time. Cheng’s scientific research had the great feature of integrating theory with practice and focusing on practical problems. After returning to China in 1934, he continued to conduct in-depth research on the nutritive value of soybean protein in close combination with the national conditions. At the same time, he carried out a series of nutritional investigations and research on food analysis, such as “Survey on the winter diet in Nanjing” (Cheng et al., 1935) and “The nutritional value of whole wheat and whole rice in regard to the growth, hemoglobin and calcium and inorganic phosphorus of the serum and bone of the albino rat” (Cheng and Tao, 1935). Aimed at the practical problems faced in improving the national nutritional status, these studies had actual effects. It is particularly worth mentioning that Cheng’s experiments on “food-combination poisoning” in animals and human beings provided substantial evidence that effectively disproved this long-rumored fallacy. In the summer of 1935, so-called poisoning by banana and yam was rumored among the folk of Nanjing and had a great influence at that time. Cheng believed that such problems, closely related to the daily life of people, should be judged and explained through scientific experiments. At first, he ate banana and yam simultaneously in order to prove that they can be eaten together without causing poisoning. Then, he collected 184 pairs of so-called poisonous food combinations from the ancient Chinese books and selected 14 pairs from them, including crab and persimmon, peanut and cucumber, and so on, which were common in the daily life. Next, the food combinations were prepared and fed to albino rats, monkeys, or dogs for two days successively, according to the usual home method. He also selected the seven most common pairs for human experiments on himself and one of his colleagues. After intake of each food combination, the expression, behavior, body temperature, and color and frequency of the excreta of the animals and human beings were observed closely for 24 h. All of the results were normal and showed no noticeable symptoms of poisoning (Cheng, 1936).

**Figure 3:**
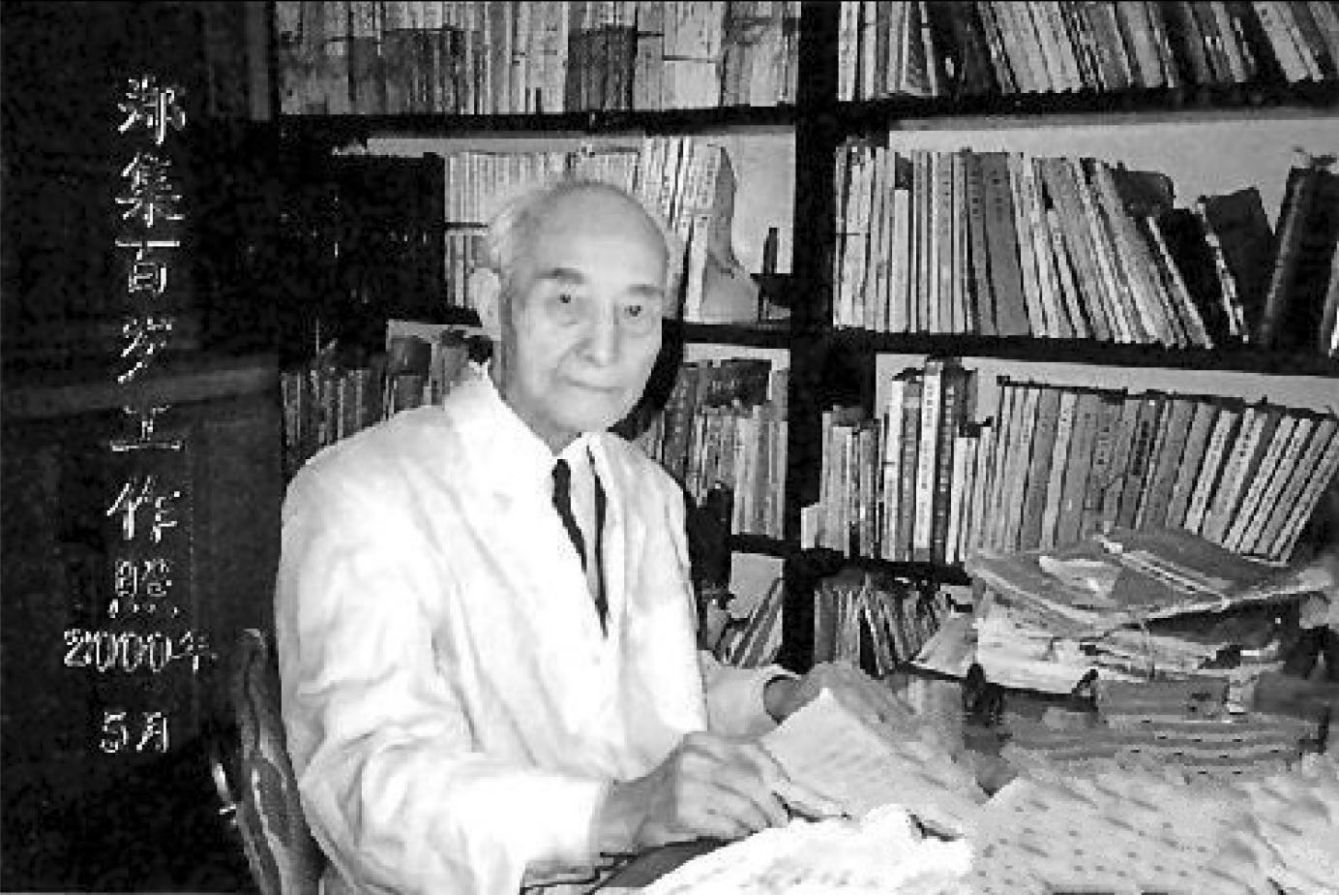
Libin T. Cheng working at his office at the age of 100

In 1974, Cheng, 74 years old, created a new direction for research into the biochemical mechanisms of aging in China. Starting with investigation and research, he surveyed more than 100 healthy people over 70 years old, analyzed the reasons for their health and longevity, and summed up 10 health and longevity experiences. In the following 10 years, he completed more than 10 papers, together with his cooperators, with regard to the correlation of aging and enzyme, nucleic acid, and other compositional changes in the red blood cells, whole brain, liver, pancreas, thymus gland, and other organs, which yielded a lot of valuable results. Based on a series of biochemical studies at the cellular and molecular levels, Cheng creatively proposed a “metabolic imbalance theory” of the aging mechanism, which laid the foundation for research into aging chemistry in China. He insisted on the combination of theory and practice, and he verified the theory with his own outstanding practice of anti-aging. At the age of 100, he was healthy and quick thinking, and he still went to work quite often at his office (Fig. 3). He is quoted as always saying “As long as I am healthy in the next year, I want to complete this and that.” (Xiao and Jin, 2000, p. 293).

Libin T. Cheng wrote numerous books, including monographs, textbooks, popular science books, and others. The English version of *A Laboratory Manual of Biochemistry*, published by Canadian Methodist Mission Press in 1938, is the first self-compiled biochemistry reference book in China (Cheng, 1938); *Applied Nutrition*, published by Chengchung Book Company in 1947, is one of the two earliest officially published nutrition monographs (Cheng, 1947); *General Biochemistry* (*Version 2*), under his general editorship, won the second prize for outstanding textbooks of the colleges and universities in China (Cheng, 1985). Just before his death, his series of popular science books *The Best Doctor is Health Preserving* was published by Jiangsu Education Publishing House, which created a last footnote for his legendary life (Cheng, 2010).
